# Galectin-3 in Heart Failure: More Answers or More Questions?

**DOI:** 10.1161/JAHA.112.004374

**Published:** 2012-10-25

**Authors:** Tariq Ahmad, G Michael Felker

**Affiliations:** Duke Clinical Research Institute, Durham, NC

**Keywords:** editorials, biomarkers, galectin-3, heart failure, renal function

“Everything is vague to a degree you do not realize till you have tried to make it precise.”

— “The Philosophy of Logical Atomism,” Bertrand Russell

Heart failure, classically defined as the heart's inability to “pump blood at a rate commensurate with the requirements of metabolizing tissue,” has long been considered a disease of the myocardium.^1^ With improved understanding of the molecular mechanisms of heart failure, it is increasingly clear that heart failure is a systemic disorder and results from the complex interplay between numerous cardiac and extracardiac pathologies.^2^ Within the clinical spectrum of heart failure, there is significant heterogeneity of phenotypic subtypes with potentially different pathophysiology, treatments, and outcomes. Given that diverse molecular pathways such as cardiac stress, fibrosis, inflammation, and injury can be quantified, biomarkers have the potential to play a critical role in the diagnosis and management of patients with heart failure, allowing better characterization of molecular mechanisms at the bedside. Galectin-3 is one such biomarker and has been shown to be predictive of adverse outcomes in patients with heart failure via mechanisms that are now being elucidated.

A PUBMED search of galectin-3 resulted in >1500 publications that span the breath of human disease, ranging from HIV infection to melanoma. This is because of the central regulatory role that galectin-3, a member of an evolutionarily conserved family of soluble β-galactoside-binding lectins, plays in several diverse biological processes and disease states.^3^ Current data suggest that galectin-3 represents a link between inflammation and fibrosis, as circulating levels have been found elevated in several human fibrotic conditions, including cirrhosis, idiopathic lung fibrosis, pancreatitis, and renal failure.^4^ In the heart, galectin-3 is thought to augment fibrosis and modulate immune response, both pivotal processes in maladaptive cardiac remodeling. A causal relationship between galetin-3 and the development of heart failure was suggested by seminal work showing that galectin-3 infusion into murine pericardium induced adverse cardiac remodeling, whereas coinfusion with an inhibitor counteracted these effects.^5^,^6^ Several studies have since shown that elevated concentrations of galectin-3 provide important prognostic information, particularly in patients with chronic heart failure.^7^–^10^

Although galectin-3 has graduated rapidly from the bench to the bedside, the mechanisms by which elevated levels influence prognosis in heart failure require further study, especially if galectin-3 is to be viewed as a “disease mediator” rather than simply a “disease marker.” In this issue of the *Journal of the American Heart Association*, Gopal and colleagues extend what is known about the association between galectin-3, the etiology and acuity of heart failure, and renal function.^11^ The authors measured galectin-3 levels in a retrospective study of 119 patients comprising these 6 cohorts: patients with acute and chronic heart failure with preserved and reduced ejection fraction, patients with no heart failure but moderate renal insufficiency, and patients with no known heart failure or renal insufficiency. Galectin-3 levels were similarly elevated in all patients with heart failure, regardless of whether it was acute or chronic or it was systolic or diastolic in nature. Whereas galectin-3 levels in heart failure patients correlated with NT-proBNP (a measure of disease acuity and severity), this relationship was significantly attenuated after consideration of age and glomerular filtration rate (GFR). Conversely, the relationship between galectin-3 level and GFR persisted after corrections for age, left ventricular ejection fraction, and NT-proBNP. This association did not vary according to the presence (*r*=−0.75, *P*<0.001) or absence (*r*=−0.82, *P*<0.001) of heart failure, as the curvilinear relationships demonstrated between galectin-3 and renal function were superimposable for patients with and without heart failure. Among patients with heart failure, estimated GFRs (eGFRs), rather than age, sex, and LVEF, were predictive of galectin-3 levels. These results suggest that the prognostic role of galectin-3 in heart failure may be related as much to renal impairment as to cardiac dysfunction.

The strong association between galectin-3 level and renal dysfunction has been previously reported in studies of patients with both acute and chronic heart failure.^9^,^10^,^12^ However, the mechanism of this relationship and its clinical implications remain uncertain: do elevated galectin-3 levels cause worsening heart failure by acting as a mediator of a dysfunctional cardiorenal axis? Data from murine models of renal failure suggest that galectin-3 plays a central role in the normal development of the kidney, as well as modulation of the inflammation, apoptosis, and fibrosis that occurs in kidney disease.^13^ Although the current study supports a renal contribution to galectin-3 elevation in patients with all forms of heart failure, we cannot confidently single out renal impairment as the primary determinant of galectin-3 elevations in heart failure on the basis of these data alone. A moderate degree of renal dysfunction is extremely common in patients with heart failure, and all heart failure patients in the current study had some degree of abnormal kidney function (eGFR 46 to 69 mL/min per 1.73 m^2^). In prior studies, the prognostic value of galectin-3 has generally persisted despite adjustment for GFR, suggesting that the influence of renal dysfunction does not preclude an important prognostic value for galectin-3.^7^,^9^,^10^ In the current study, the primary etiologies of chronic kidney disease in patients without heart failure were hypertension and diabetes, both strong risk factors for cardiovascular disease. It is thus possible that galectin-3 elevation in these patients reflects subclinical cardiovascular disease rather than abnormal renal function alone.^14^ Future studies that more carefully characterize cardiac and renal abnormalities even in the absence of clinically overt disease will be required to fully disentangle the relationship among galectin-3 level, renal function, and heart failure.

Do the current results shed light on the mechanism of galectin-3 elevation in heart failure? Given the role of galectin-3 in a diverse set of pathophysiological conditions, ranging from inflammatory bowel disease to stroke, it is likely that elevations of galectin-3 in heart failure patients signify an incompletely understood systemic inflammatory and profibrotic response. Whether this adverse biochemical milieu potentiates end-organ injury caused by known risk factors such as hypertension and diabetes or directly causes it remains to be clarified. The relationship with renal impairment shown in the current study may be a single piece of a much larger puzzle.

How do these findings inform clinical use of galectin-3 in heart failure? To address this question, we must view galectin-3 within the broader framework of how biomarkers are used in cardiovascular disease. Biomarkers can be used for diagnosis (eg, troponin), to provide prognostic information (eg, natriuretic peptides), and to influence selection of treatment strategies (eg, C-reactive protein).^15^ This study supports the general concept that galectin-3 lacks the sensitivity and discrimination required for making a diagnosis of heart failure.^16^ Galectin-3 levels can help with quantification of absolute risk in heart failure; however, exactly how the data should be interpreted in the face of established clinical and risk markers such as the natriuretic peptides is currently unclear.^7^,^10^ Also, the strong association of galectin-3 level with the degree of renal dysfunction shown in the current study raises the question of whether different prognostic cut points of galectin-3 should be used for patients with renal disease. Finally, there is some data to suggest that galectin-3 may play an important role in delineating patients who may benefit from therapies versus those who may not: among patients with chronic heart failure who were enrolled in the CORONA trial, low levels appeared to identify a subgroup that benefited from rosuvastatin.^17^ Current treatments for heart failure, although effective, are expensive and may cause an unacceptably high rate of adverse events when applied broadly. This study reveals the significant disconnect between biochemical measures of heart failure (galectin-3) and common clinical measures (left ventricular ejection fraction), implying a need for further investigations of galectin-3 and treatment effect in most therapeutic areas in heart failure, where outcomes may be improved with “biological” guidance of therapies.

Continued investigations into the relationship between galectin-3 and heart failure appear to have led to more questions than answers. Moving forward, well-designed studies are needed to answer some key questions. Is galectin-3 a marker or mediator of worsening heart failure? Why are galectin-3 levels elevated in renal disease, and do they contribute to continued renal impairment? How do the prognostic consequences of galectin-3 elevations differ in heart failure patients with and without concomitant renal dysfunction? Can galectin-3 levels identify patients who may or may not derive benefit from specific heart failure therapies? Can pharmacological blockage of galectin-3 attenuate decline in cardiac or renal function? Although much about the details of galectin-3 in heart failure remains vague, continued efforts at increasing precision may uncover a new chapter in our understanding of heart failure pathophysiology.
